# Human amnion mesenchymal stem cells restore spermatogenesis in mice with busulfan-induced testis toxicity by inhibiting apoptosis and oxidative stress

**DOI:** 10.1186/s13287-020-01803-7

**Published:** 2020-07-16

**Authors:** Chunfeng Qian, Qingxia Meng, Jiafeng Lu, Liya Zhang, Hong Li, Boxian Huang

**Affiliations:** 1 Center of Reproduction and Genetics, Affiliated Suzhou Hospital of Nanjing Medical University, Suzhou Municipal Hospital, Suzhou, 215002 China; 2 State Key Laboratory of Reproductive Medicine, Nanjing Medical University, Nanjing, 210029 China

**Keywords:** Human amnion mesenchymal stem cells, Spermatogenesis, Busulfan, Reactive oxygen species

## Abstract

**Background:**

Before starting gonadotoxic therapies, cryopreservation of mature sperm has been proposed worldwide as a method for male fertility preservation and for enabling the conception of a healthy baby with assisted reproductive technology (ART); however, these technologies are not feasible for prepubertal boys and men with spermatogenic failure. Transplantation of mesenchymal stem cells has exhibited successful therapeutic benefits in restoring spermatogenesis via gonadal graft angiogenesis, transplanted cell clonogenesis, and disordered somatic compartment recovery. This study aimed to elucidate the fertility protective effects and the underlying mechanisms of human amnion mesenchymal stem cells (hAMSCs) against busulfan-induced testis toxicity.

**Methods:**

An in vivo busulfan-induced testis toxicity mouse model and an in vitro busulfan-administered mouse Sertoli cell line were employed to evaluate the efficacy and mechanisms of hAMSC transplantation on male fertility preservation. The process of spermatogenesis was evaluated histologically, and the percentage of seminiferous tubules with vacuoles was evaluated by HE staining. Semen parameters were calculated by computer-assisted semen analysis. ELISA was employed to test the testosterone concentration and the levels of oxidative- and antioxidative-associated substances LDH, MDA, GR, SOD, GPx, and CAT. The rates of proliferation (Ki67), apoptosis (Annexin V), and ROS were measured by FACS. The fluorescence intensity of a marker of apoptosis (TUNEL) and a meiosis gene in spermatogenesis (SCP3) were detected by immunofluorescence assay. The expression of mRNA in germ cell-specific (GCS) genes (Dazl, Ddx4, and Miwi) and meiosis genes (Scp3, Cyclin A1, and Stra8) was tested by qPCR. The expression of antiapoptotic proteins (SURVIVIN and BCL2), apoptotic proteins (CASPASE3 and CASPASE9), GCS proteins (Dazl, Ddx4, and Miwi), and meiosis proteins (Scp3, Cyclin A1, and Stra8) was tested by western blotting.

**Results:**

hAMSC transplantation following disruption by busulfan-induced testis toxicity restored spermatogenesis, elevating testosterone levels and enhancing testicular weight, size, and semen parameters in vivo. In addition, hAMSCs clearly ameliorated cell apoptosis, enhanced cell proliferation, repressed oxidative damage, and augmented oxidative defense in vivo and in vitro. Moreover, hAMSCs distinctly increased the expression of the GCS genes Dazl, Ddx4, and Miwi and the meiosis genes Scp3, Cyclin A1, and Stra8 in vivo.

**Conclusions:**

hAMSCs might represent a promising tool for the use in regenerative medicine, as these cells can restore spermatogenesis in a busulfan-induced testis toxicity mouse model and facilitate activity in a busulfan-administered mouse Sertoli cell line by resisting apoptosis and oxidative stress.

## Background

Mammalian spermatogenesis is a precise process in which haploid spermatozoa are generated from diploid spermatogonia. It proceeds first through mitosis, then meiosis, and finally spermiogenesis, and it involves the coordination of numerous genes [1]. A slightly awry deviation at any of these highly regulated processes can result in male infertility [2]. Approximately 15% of the couples fail to fertility, and male factors are involved in half [3]. Although many factors, such as genetics, hormonal disorders, psychological stress, sex problems, obesity, medications, and substances, as well as a variety of unknown etiologies, may contribute to male infertility, the failure to produce sperm is the main manifestation of severe oligospermia and azoospermia which exhibits male infertility phenotypes [4–6].

Many hopelessly infertile couples with male severe oligospermia can become parents via the development of assisted reproductive technologies (ART); however, these technologies, which are relatively expensive and not available for azoospermic failures of spermatogenesis, may use genetically defective sperm in fertilization by using technology to traverse natural barriers. Although the promising survival rate of childhood cancers who may receive radiotherapy and certain chemotherapies, their fertility is impaired when they enter reproductive age [7]. In a cohort of more than 10 years of follow-up observation among 214 survivors of childhood cancers with alkylating agent chemotherapy, azoospermia was observed in 25% of patients, and oligospermia was observed in 28% [8]. Therefore, it is an urgent matter to explore an efficient and safe approach to preserve male future reproductive capacity. Taking into account that immature testicular tissue or spermatogonial stem cells for prepubertal malignancy boys autotransplanted when they want to be fathers may be contaminated with malignant cells and change epigenetic modifications after long-term cryopreservation or propagation, mesenchymal stem cell (MSC) therapy has exhibited a gratifying therapeutic effect on rehabilitating the endogenous spermatogenesis microenvironment for male fertility preservation [9].

MSCs derived from the umbilical cord (UCMSCs) [10], bone marrow (BMMSCs) [11], and niche cells [12] can be transplanted directly into the testis of a busulfan-induced gonad toxicity animal model to improve spermatogenesis. Recently, conditioned media from BMMSCs [13] and exosomes from urine-derived stem cells [14] facilitated spermatogenesis impaired by busulfan-induced testis toxicity in mice by alleviating apoptosis in spermatogenic cells, promoting intercellular adhesion molecules, and upregulating spermatogenesis-associated genes. Furthermore, several studies demonstrated that an in vitro coculture system using human umbilical cord-derived MSCs seeded on monolayer mouse Sertoli cells [15] and using BMMSCs with an upper filter of testicular cell suspension [16] also increased the expression of male germ cell markers. Although the above MSCs in vivo or in vitro showed effectiveness in rescuing impaired spermatogenesis, whether hAMSCs also have therapeutic effects in terms of improving testicular fertility preservation is not clear.

hAMSCs possess some remarkable features, such as low immune rejection, low inflammatory activity, ease of noninvasive harvesting, and minimal ethical issues, which make them suitable as a potential regenerative medicine [17]. A previous study showed that hAMSCs restore the natural aging of the ovary, contributing to the secretion of growth factors [18]. In addition, hAMSCs exerted more effective reproductive protection for impaired ovaries induced by cyclophosphamide than human amnion epithelial cells due to the superior telomerase and pluripotent activity and the different secretory soluble factors [19]. Moreover, earlier research revealed that hAMSCs induced by bone morphogenetic protein 4 and retinoic acid differentiated into germ cells and exhibited Itgb1, Dazl, Stra8, Piwil2, Mvh, c-Kit, and Dazl [20].

Even though several MSCs exert a promising therapeutic benefit to recover dysfunctional cells, tissues, or organs, the underlying fundamental aspects of how stem cells contribute to homeostasis and repair are not elaborated clearly. The physiological level of reactive oxygen species (ROS) is closely related to spermatogenesis; however, increased concentrations of ROS overwhelm the antioxidant scavenging system, resulting in oxidative stress (OS) [21]. Due to the greater degree of compaction of mammalian sperm DNA and limited DNA-damaging defense and repair systems, spermatozoa are highly sensitive to OS [22]. ROS-mediated spermatozoa DNA damage is a main reason for male infertility [23]. A previous study verified that hAMSCs demonstrated a powerful potential to ameliorate OS in BMMSCs induced by H_2_O_2_ [24]. Moreover, another study confirmed that BMMSCs effectively enhanced antioxidant enzymes and decreased the percentage of DNA fragmented in spermatozoa to restore spermatogenesis [25].

The male reproductive protection role of hAMSCs in busulfan-induced testis toxicity mice and in busulfan-treated mouse Sertoli cells have not been explored, so the present study focused on the effectiveness and the underlying mechanism of hAMSCs in restoring spermatogenesis.

## Methods

### hAMSC preparation

An hAMSC line was established, and the characteristics were verified as previously described [18]. In brief, hAMSCs were seeded in six-well plates at a density of 1 × 10^5^ cells/well in DMEM (Gibco, USA) supplemented with 10% FBS (Gibco, USA), L-glutamine (Gibco, USA), penicillin/streptomycin (Gibco, USA), bFGF (R&D, USA), and EGF (R&D, USA), and they were grown in a standard incubator. hAMSCs at passages 3–5 were employed for the experiments in this study.

### Sertoli cell preparation and treatment

The TM4 (Procell, China) mouse Sertoli cell line was purchased and cultured at a density of 1 × 10^5^ cells per well in six-well plates using TM4 special complete medium (Procell, China) in a standard incubator. Cell medium was changed every 2 days, and cells were trypsinized and passaged when at 80–90% confluence. Busulfan was administered to Sertoli cells with some modifications according to the method described previously [26]. Briefly, busulfan at a concentration of 10^−4^ μM was added to the Sertoli cells, and they were incubated for 48 h in a standard incubator. Then, a transwell system was applied to coculture the hAMSCs (upper inserts) with the busulfan-administered Sertoli cells (bottom of six-well plates) for 48 h. Thereafter, three groups were assigned to Sertoli cells: the control group (no treatment), the BSF group (busulfan treatment), and the BSF/hAMSC group (busulfan treatment followed by hAMSCs).

### Experimental animals

The protocol of acquisition and processing animals was implemented in accordance with the Animal Care and Use Committee of Nanjing Medical University. Nanjing Medical University offered male C57BL/6 mice (6–8 weeks old, total number is 150). They were fed under standard conditions as described in our previous research [18]. A busulfan-induced testis toxicity mouse model was established following the method described previously with some modifications [10]. In brief, DMSO was used to dissolve busulfan, and distilled water was used to dilute the busulfan to a concentration of 5 mg/ml. Busulfan (40 mg/kg) was injected into mice intraperitoneally. After busulfan administration for 4 weeks, a single dose of approximately 1 × 10^7^ hAMSCs suspended in normal saline was injected into the testes. Subsequently, three groups were assigned to mice: the control group (no treatment, *n* = 50), the BSF group (treatment with busulfan, *n* = 50), and the BSF/hAMSC group (treatment with busulfan followed by hAMSCs, *n* = 50).

### Testes measurement and histological analysis

Nine 8-week-old mice were assigned to three groups (*n* = 3 per group). The control group received no treatment for 5 weeks, the BSF group received no treatment for 4 weeks and then BSF treatment for 1 week, and the BSF/hAMSC group received BSF treatment for 4 weeks and then hAMSC treatment for 1 week. Testes and blood were obtained for HE staining and ELISA, respectively. Eight-week-old mice were assigned to three groups (*n* = 47 per group). The control group received no treatment for 8 weeks, the BSF group received no treatment for 4 weeks and then BSF treatment for 4 weeks, and the BSF/hAMSC group received BSF treatment for 4 weeks and then hAMSCs treatment for 4 weeks. All groups were euthanized at the same time, and the testes, epididymides, and blood were harvested for the following experiments. For the testes obtained from all the mice, the weight, length, and width were measured. Paraformaldehyde was used to fix the testes of the three groups after hAMSC transplantation; then, the tissues were dehydrated, xylene clarified, and paraffin embedded, and the paraffin-embedded blocks were cut to produce 5-μm-thick sections; the sections were hematoxylin-eosin stained, and slides were analyzed with a light microscope as described in a previous study [18]. The seminiferous tubules with vacuoles were counted in five representative sections of each testis.

### TUNEL assay and SCP3 immunofluorescence

Paraffin sections of the testes from the three groups following hAMSC transplantation were used for TUNEL assay and SCP3 immunofluorescence. A TUNEL Assay Kit (Abcam, USA) was employed to detect DNA fragmentation according to the manufacturer’s directions. In brief, the slides were deparaffinized with xylene, rehydrated with isopropanol, incubated with a proteinase K solution, refixed with formaldehyde, and incubated in a DNA labeling solution and an antibody solution. The same slides that were counterstained with an anti-SCP3 antibody (Abcam, USA) and then were fixed with 4% PFA (Sigma, USA); then, the slides were permeated using 0.1% Triton X-100 (Sigma, USA), blocked using 4% bovine serum albumin (BSA; Sigma, USA), incubated with an anti-SCP3 antibody (Abcam, USA), and subjected to TUNEL staining at 4 °C overnight. Slides were then stained using FITC-conjugated secondary antibodies (Jackson Immunoresearch, West Grove) before being mounted with Hoechst 33342 (Beyotime Biotechnology, China) and analyzed with a fluorescence microscope (Olympus, Japan).

### Computer-assisted semen analysis

The epididymal tails were gathered from the three groups of mice following hAMSC transplantation to analyze the seminal parameters according to the manufacturer’s instructions. In brief, the epididymal tail was excised, placed in HTF (EasyCheck, China), and incubated in a metal bath for 5 min at 37 °C. The total number of sperm and the percentage of static sperm and rapidly moving sperm were analyzed by computer-assisted semen analysis (CASA).

### ELISA analysis

Blood from the mice in the three groups was obtained for ELISA analysis of the levels of testosterone, lactate dehydrogenase (LDH), malondialdehyde (MDA), glutathione reductase (GR), superoxide dismutase (SOD), glutathione peroxidase (GPx), and catalase (CAT). The TM4 special conditioned medium from the three groups of Sertoli cells was assessed for the levels of LDH, MDA, GR, SOD, GPx, and CAT via ELISA analysis with an ELISA kit (Cayman Chemical, USA). Briefly, 50 μl of serum or medium was prepared on the test plate and incubated for 30 min at 37 °C; then, the wells were washed for 10 s five times, and 50 μl of HRP-conjugate reagent was added and incubated with the cells for 60 min at 37 °C again. Then, the wells were washed for 10 s five times and incubated for 30 min at 37 °C with 50 μl of a mixture of substrate A and B solutions; finally, 50 μl of stop solution was added to the wells. Ultimately, the light absorbance was detected by using a spectrophotometer (BioTek, USA).

### Fluorescence-activated cell sorting (FACS) analysis

Sertoli cells were harvested to detect Ki67, Annexin V, and ROS, and the testes from the three groups were digested to generate single cells using 0.25% trypsin-EDTA. A Fixation/Permeabilization Solution Kit (BD, USA) was applied to fix and permeabilize the above cells. PE-conjugated anti-Ki67 (BD, USA), FITC-conjugated anti-Annexin V (BD, USA), PE-conjugated anti-ROS (Abcam, USA), and their corresponding isotypes were labeled for 30 min at 4 °C. Then, flow cytometry (Beckman, USA) was employed for analysis according to the manufacturer’s instructions.

### Western blot analysis

The mouse testes of the three groups after hAMSC treatment and the Sertoli cells of the three groups were harvested for western blotting as described previously [18]. Antibodies for BCL2, SURVIVIN, CASPASE9, CASPASE3, and GAPDH, which were obtained from Abcam (USA), were used for Sertoli cells. Antibodies for BCL2, SURVIVIN, CASPASE9, CASPASE3, Dazl, Ddx4, Miwi, Scp3, Cyclin A1, Stra8, GAPDH, and β-Actin were obtained from Abcam (USA) and were used for the testes.

### RNA extraction and quantitative real-time polymerase chain reaction (qPCR)

Total RNA was extracted from the testes of the three groups of mice after hAMSC treatment by using a QIAGEN RNeasy Mini Kit (QIAGEN, USA). A PrimeScript RT Reagent Kit was used to reverse-transcribe cDNA, SYBR Premix Ex Taq (Takara, Japan) was employed to implement quantitative real-time polymerase chain reaction (PCR) using a Thermal Cycler Dice Real Time System (Takara, Japan), and the 2-ΔΔCt calculation method was performed to parse the data following the manufacturer’s methods. The primer sequences of the genes Dazl, Ddx4, Miwi, Scp3, Cyclin A1, Stra8, and the internal reference (GAPDH) are shown in supplementary Table 1.

### Statistical analysis

All experiments in the present study were repeated a minimum of 3 times. The data are represented as the mean ± SD. One-way ANOVA was performed using SPSS 21.0 software to analyze significant differences, which were defined as having a *P* value of less than 0.05.

## Results

### hAMSCs recovered impaired spermatogenesis and elevated testosterone levels in a busulfan-induced testis toxicity mouse model

**Fig. 1:**
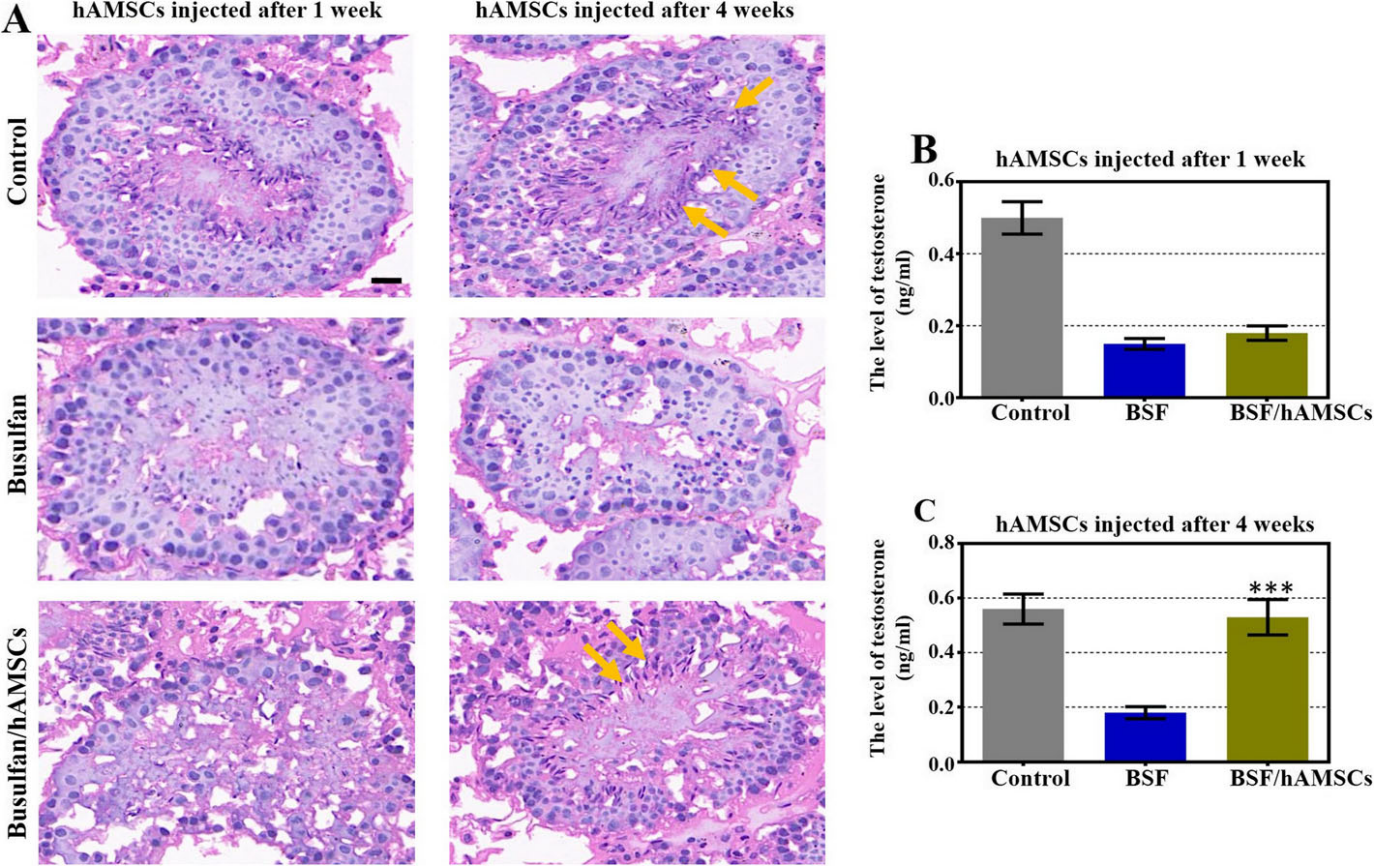
Human amnion mesenchymal stem cells (hAMSCs) restored spermatogenesis and elevated testosterone levels in a busulfan-induced testis toxicity mouse model. **a** Micrographs of mouse testis sections were obtained by HE staining in three groups 1 week and 4 weeks after hAMSC transplantation. Scale bar = 5 μm. *n* = 10 for each group. **b** Testosterone expression was tested by ELISA analysis of the three groups 1 week after hAMSC transplantation. **c** Testosterone expression was determined by ELISA analysis of the three groups 4 weeks after hAMSC transplantation. The results are presented as the mean ± SD. ***p < 0.001 (compared to the BSF group). *n* = 10 for each group

To explore the possible therapeutic benefits of hAMSCs in restoring spermatogenesis that had been disrupted by busulfan treatment, we evaluated the phenotype of the seminiferous tubules and the testosterone level in the three groups by HE staining and ELISA. As shown in Fig. 1a, typical morphology indicating complete spermatogenesis was observed in the control group, while all of the healthy sperm and the round spermatids disappeared with the expansile lumen after busulfan treatment for 1 week. Further, the majority of spermatogonia and almost all of the primary spermatocytes, secondary spermatocytes, round spermatids, and healthy sperm were absent from the seminiferous tubules, and there was obvious vacuolation in the basement membrane after busulfan treatment for 4 weeks. At this time, a complete spermatogenetic arrest mouse model was established. The appearance of spermatogenetic cells in the hAMSC-transplanted group at week 1 was similar to that of the BSF group. However, the location of cells at different stages of spermatogenesis was reappeared, and the lumen had gotten smaller at 4 weeks after hAMSC transplantation. Compared to the BSF group, the testosterone expression level of the hAMSC-transplanted group at week 1 was slightly improved, while it was obviously enhanced at 4 weeks after hAMSC transplantation (Fig. 1b, c).

### hAMSCs improved testicular weight, size, and semen parameters in a busulfan-induced testis toxicity mouse model

**Fig. 2:**
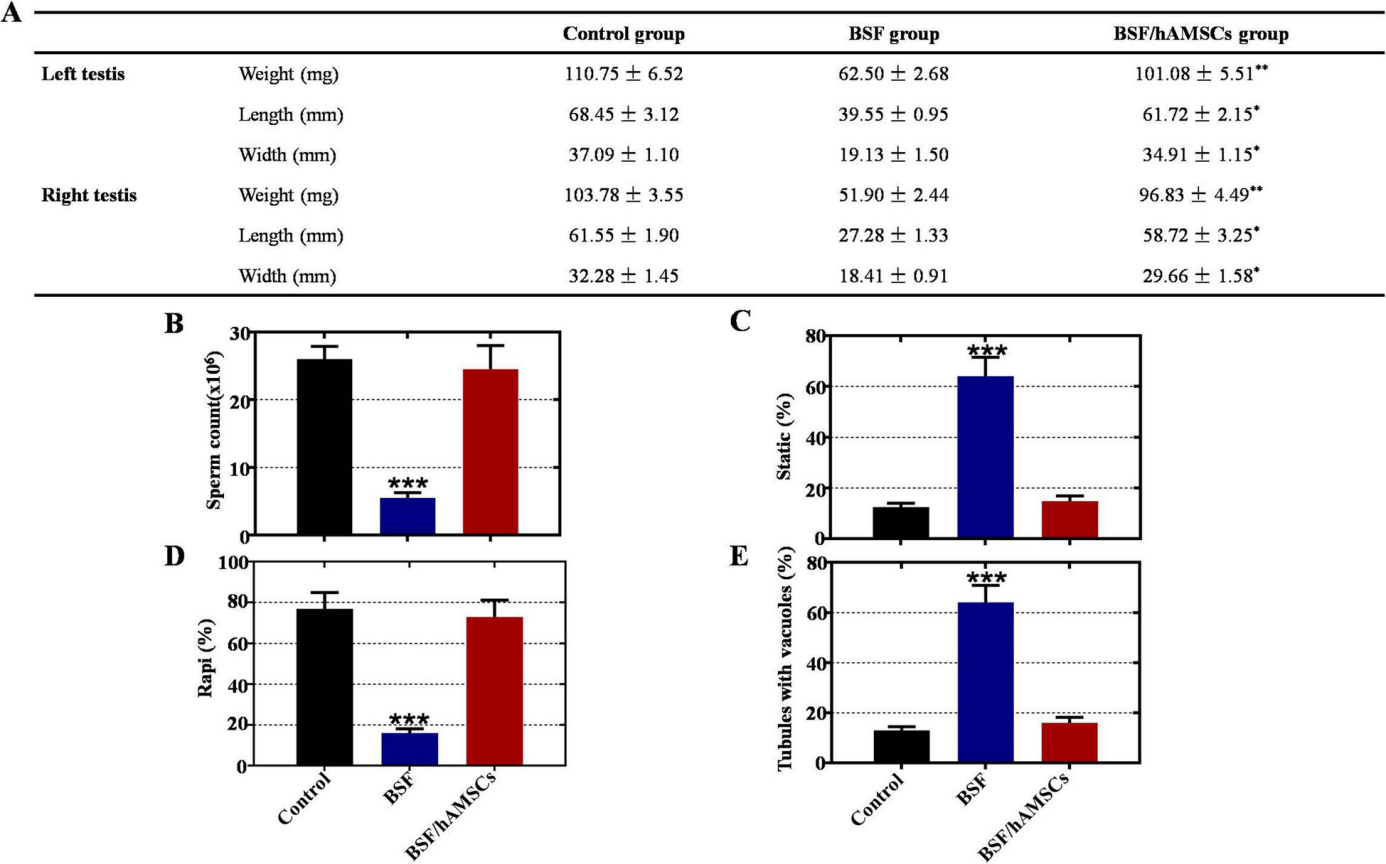
hAMSCs improved testicular weight, size, and semen parameters in a busulfan-induced testis toxicity mouse model. **a** Changes in mouse testicular weight, length, and width in the three groups after hAMSC transplantation. **b**–**d** The sperm count, static sperm proportion, and rapidly moving sperm proportion were calculated by CASA tests of the three groups after hAMSC transplantation. **e** The proportion of seminiferous tubules with vacuoles among the three groups was counted using HE-stained sections of mouse testis after hAMSC transplantation. The results are presented as the mean ± SD. *p < 0.05, **p < 0.01, and ***p < 0.001 (compared to the BSF/hAMSC group)

To better understand whether hAMSCs restore testis function, we compared testicular weight and size among the three groups. In comparison with the control group, the testicular weight, length, and width were reduced by approximately 50% in the BSF group. However, the same parameters were remarkably enhanced to near normal levels at 4 weeks after hAMSC transplantation (Fig. 2a). Sperm parameters, as assessed by the CASA test, were calculated to further determine whether hAMSCs restored spermatogenesis function. Compared to the control group, a more than five-fold decline in the total number of sperm was detected in the BSF group, while it recovered to near normal levels at 4 weeks after hAMSC injection (Fig. 2b). The proportion of rapidly moving sperm decreased dramatically in the BSF group when compared with the control group, and it was obviously enhanced in the BSF/hAMSC group compared with the BSF group (Fig. 2c). At week 4 after hAMSC transplantation, an approximate five-fold decrease in the proportion of static sperm was observed in comparison with that of the BSF group (Fig. 2d). The seminiferous tubules with vacuoles were counted, and the results indicated that the proportion of seminiferous tubules with vacuoles was significantly decreased in the BSF/hAMSC group versus the BSF group (Fig. 2e).

### hAMSCs enhanced cell proliferation and attenuated busulfan-induced damage in Sertoli cells

**Fig. 3:**
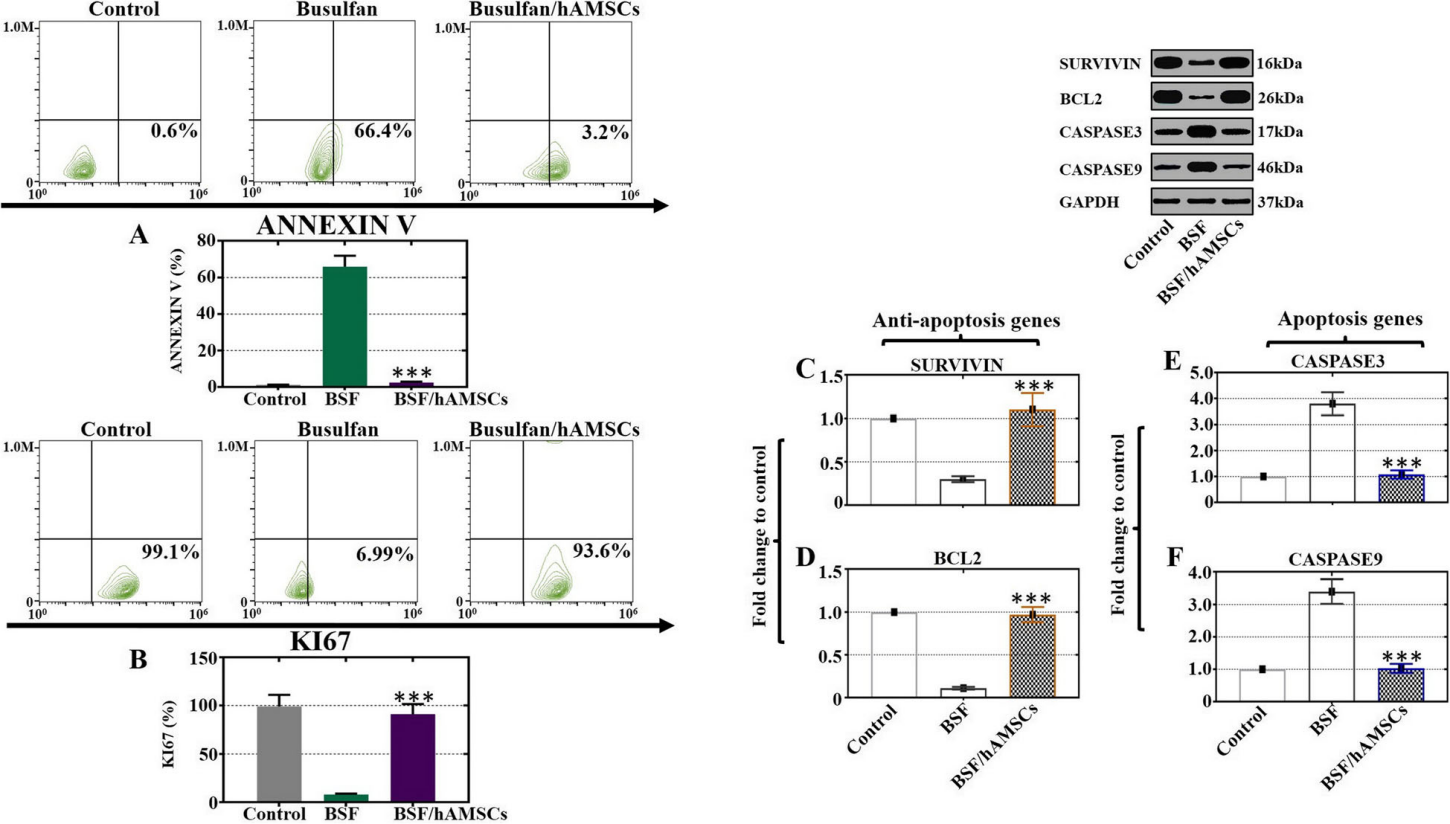
hAMSCs enhanced cell proliferation and attenuated busulfan-induced damage in Sertoli cells. **a**, **b** The expression levels of Annexin V and Ki67 in Sertoli cells were measured by FACS in the three groups. **c**–**f** The protein expression levels of SURVIVIN, BCL2, CASPASE3, and CASPASE9 in Sertoli cells were detected by western blot in the three groups. The results are presented as the mean ± SD. ***p < 0.001 (compared to the BSF group)

It has been convincingly shown that the imbalance of cell proliferation and apoptosis contributes to abnormal homeostasis, which affects spermatogenesis dysfunction [27]. We established an in vitro model in which mouse Sertoli cells were administered busulfan, and then, hAMSCs were cocultured with the Sertoli cells. FACS analysis was adopted to assess the ratio of the signal for the cell proliferation marker Ki67 and the signal for the cell apoptosis marker Annexin V in the three groups. The results illustrated that the rate of Annexin V was clearly increased in the BSF group compared to that of the control animals, and it was obviously suppressed in the BSF/hAMSC group compared to that of the busulfan-treated animals (Fig. 3a). Contrary to the apoptosis results, the rate of Ki67 was significantly decreased in the BSF group, but the rate was similar between the BSF/hAMSC group and the control group (Fig. 3b). The protein levels of the anti-apoptotic markers SURVIVIN and BCL2 and the apoptotic markers CASPASE3 and CASPASE9 were tested in the three groups by western blot. The results showed that the expression levels of SURVIVIN and BCL2 were significantly upregulated in the BSF/hAMSC group versus the BSF group (Fig. 3c, d). Nevertheless, the expression levels of CASPASE3 and CASPASE9 were obviously downregulated at 4 weeks after hAMSC treatment when compared to the BSF group (Fig. 3e, f).

### hAMSCs ameliorated apoptosis and improved cell proliferation and spermatocyte meiosis in a busulfan-induced testis toxicity mouse model

**Fig. 4:**
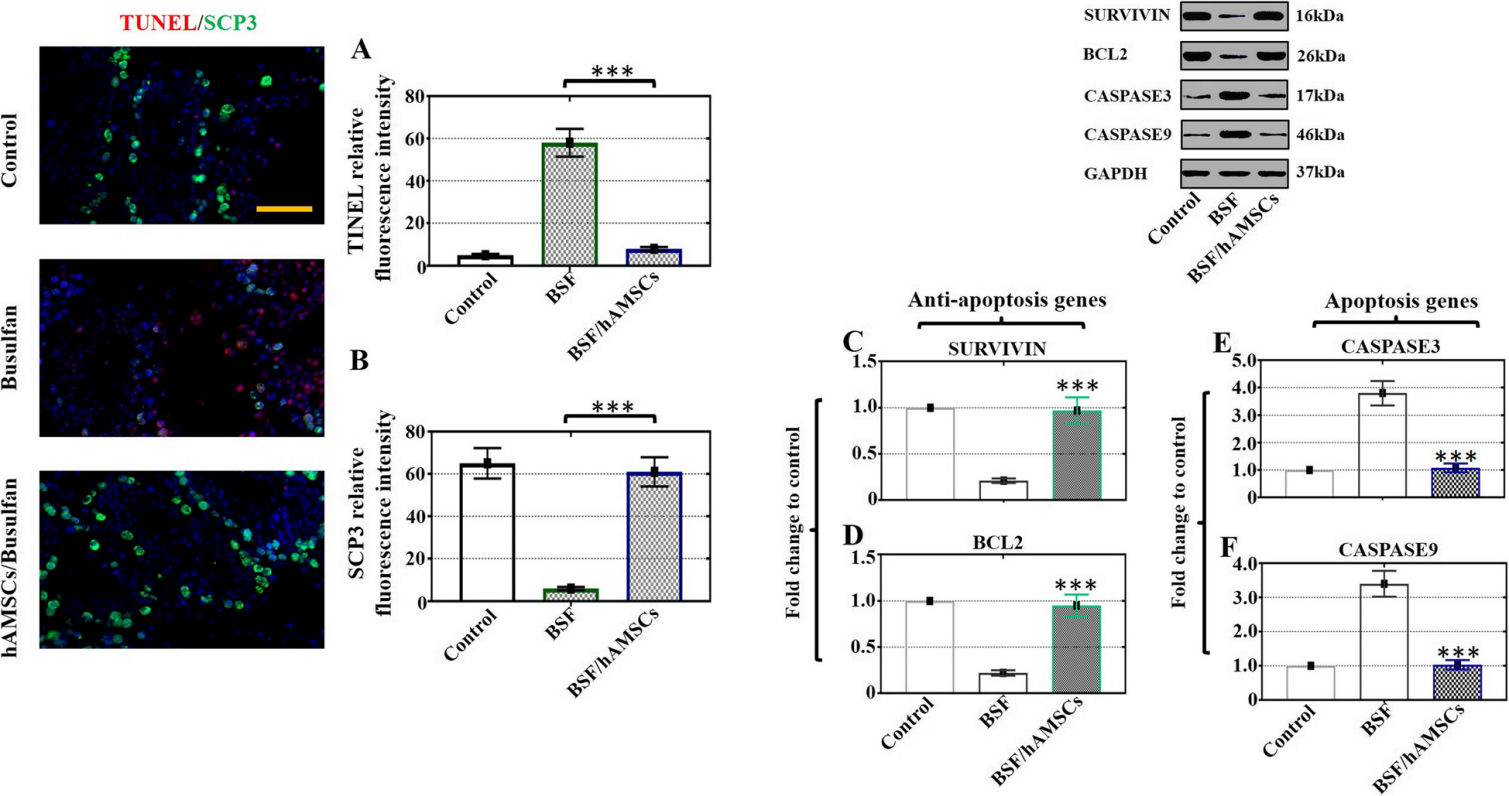
hAMSCs ameliorated cell apoptosis and improved cell proliferation and spermatocyte meiosis in a busulfan-induced testis toxicity mouse model. **a**, **b** Fluorescence microscopy was employed to detect the relative fluorescence intensity of TUNEL (red) and SCP3 (green) in the three groups. **c**–**f** The protein expression of SURVIVIN, BCL2, CASPASE3, and CASPASE9 was evaluated in the three groups by western blot. The results are presented as the mean ± SD. ***p < 0.001 (compared to the BSF group). *n* = 10 for each group

Given that hAMSCs enhanced cell proliferation and attenuated apoptosis in vitro, we used a busulfan-induced testis toxicity mouse model to evaluate testicular cell proliferation and apoptosis function following treatment with hAMSCs transplantation. As expected, hAMSCs effectively inhibited the relative fluorescence intensity of TUNEL staining and obviously augmented the relative fluorescence intensity of SCP3 compared with the BSF group, as indicated in Fig. 4a, b. In line with the in vitro results, the western blot results demonstrated an increased trend in the protein levels of SURVIVIN and BCL2 in the BSF/hAMSC group versus the BSF group (Fig. 4c, d). In comparison with those of the BSF group, the CASPASE3 and CASPASE9 protein levels exhibited a decreasing trend in the BSF/hAMSC group (Fig. 4e, f).

### hAMSCs repressed oxidative damage and augmented oxidative defense in Sertoli cells treated with busulfan

**Fig. 5:**
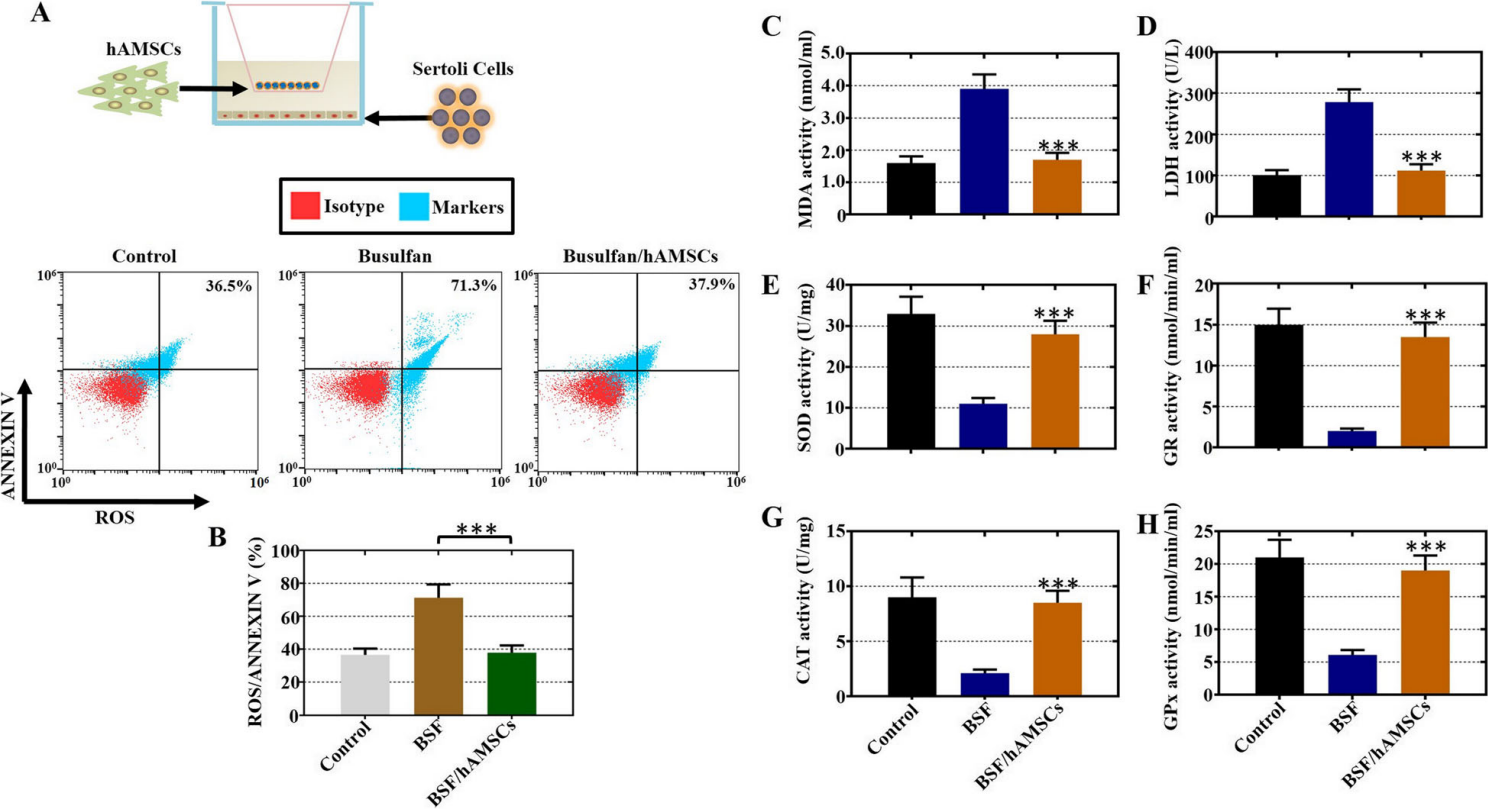
hAMSCs repressed oxidative damage and augmented oxidative defense in Sertoli cells with busulfan administration. **a** A schematic diagram of hAMSCs (on the upper coculture inserts) cocultured with mouse Sertoli cells (on the bottom of a 6-well culture plate). **b** The percentage of ROS^+^/Annexin V^+^ Sertoli cells was analyzed by FACS in the three groups. **c**–**h** The expression of LDH, MDA, GR, SOD, GPx, and CAT was determined by ELISA analysis in three groups. The results are presented as the mean ± SD. ***p < 0.001 (compared to the BSF group)

Sperm DNA can be damaged by oxidative stress that exceeds normal physiological levels, which can result in male infertility [28]; thus, we hypothesized that the hAMSC-transplanted recovery of spermatogenesis function could be an underlying stimulus by resisting oxidative stress. We employed mouse Sertoli cells with hAMSCs using a coculture system to detect the ROS rate by FACS (Fig. 5a). Our results revealed that there was a significant upregulation in the percentage of ROS^+^/Annexin V^+^ Sertoli cells (71.3%) in the BSF group; further, a significantly downregulated percentage of ROS^+^/Annexin V^+^ Sertoli cells (37.9%) was detected after hAMSC exposure, which was similar to the percentage in the control cells (36.5%) (Fig. 5b). Furthermore, the levels of LDH and MDA (oxidoreductases) and GR, SOD, GPx, and CAT (antioxidases) were analyzed by ELISA. Compared to the BSF group, hAMSC administration led to obviously decreased levels of LDH and MDA (Fig. 5c, d). Conversely, remarkably increased levels of GR, SOD, GPx, and CAT were also detected in the BSF/hAMSC group versus the BSF group (Fig. 5e–h).

### hAMSCs decreased oxidative damage and increased oxidative defense in a busulfan-induced testis toxicity mouse model

**Fig. 6:**
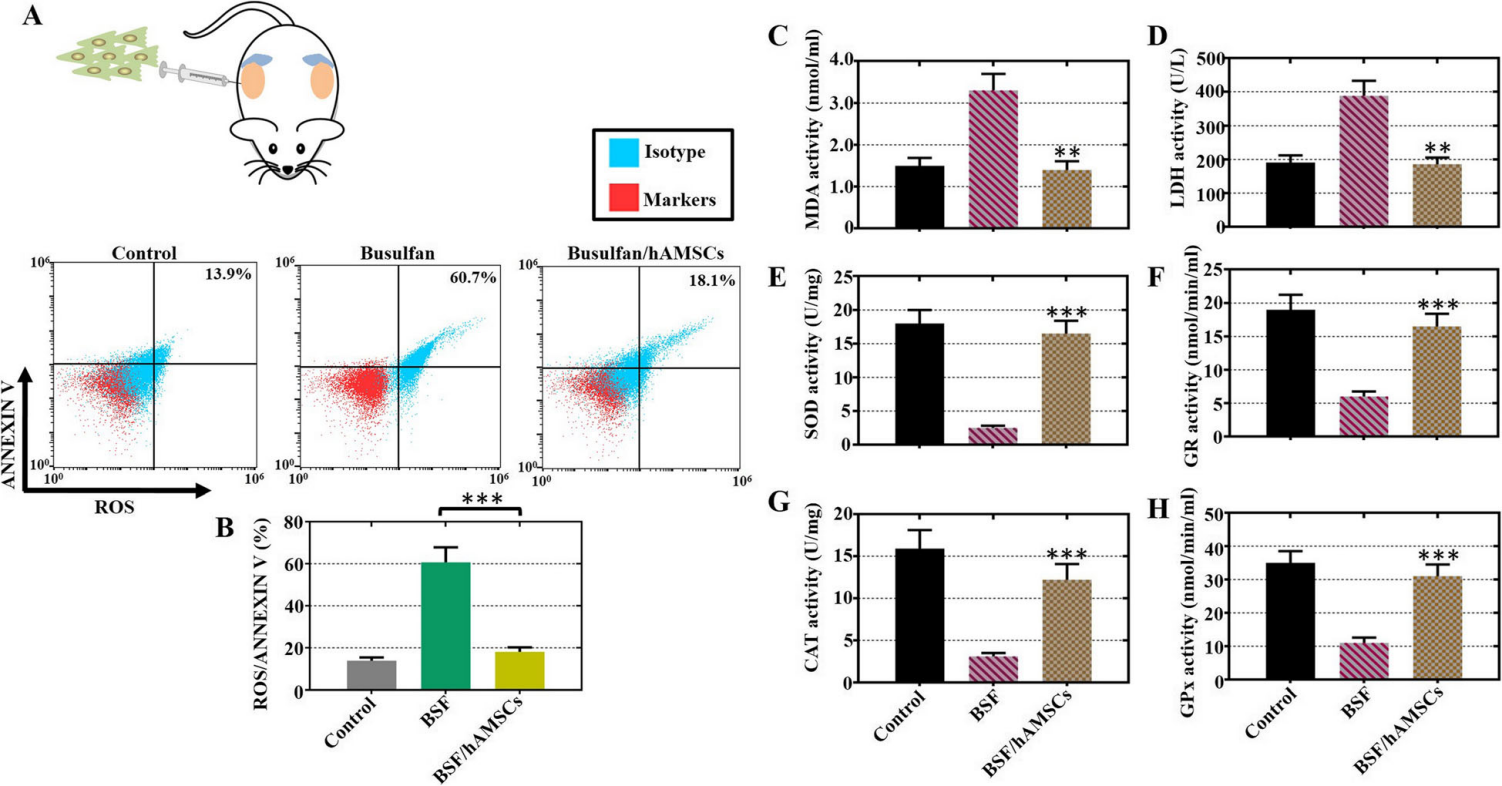
hAMSCs decreased oxidative damage and increased oxidative defense in a busulfan-induced testis toxicity mouse model. **a** A schematic diagram of hAMSCs transplanted into busulfan-induced testis toxicity mouse testes by testicular injection. **b** FACS analysis was used to measure the percentage of ROS^+^/Annexin V^+^ Sertoli cells in the three groups. **c**–**h** The expression of LDH, MDA, GR, SOD, GPx, and CAT was determined by ELISA analysis in the three groups. The results are presented as the mean ± SD. **p < 0.01, and ***p < 0.001 (compared to the BSF group). *n* = 10 for each group

Taking into account that in vitro hAMSCs repressed oxidative damage and augmented oxidative defense, a busulfan-induced testis toxicity mouse model was employed to indicate that hAMSCs restored spermatogenic function by repressing oxidative stress in vivo (Fig. 6a). On the one hand, the results of FACS showed that the percentage of ROS^+^/Annexin V^+^ Sertoli cells was apparently attenuated after the transplantation of hAMSCs (18.1%) compared to that in the BSF group (60.7%) (Fig. 6b). On the other hand, the levels of LDH and MDA exhibited an upward trend, and the levels of GR, SOD, GPx, and CAT exhibited a downward trend after hAMSC treatment for 4 weeks compared with those in the BSF group (Fig. 6c–h).

### hAMSCs elevated spermatogenesis-associated gene markers in a busulfan-induced testis toxicity mouse model

**Fig. 7:**
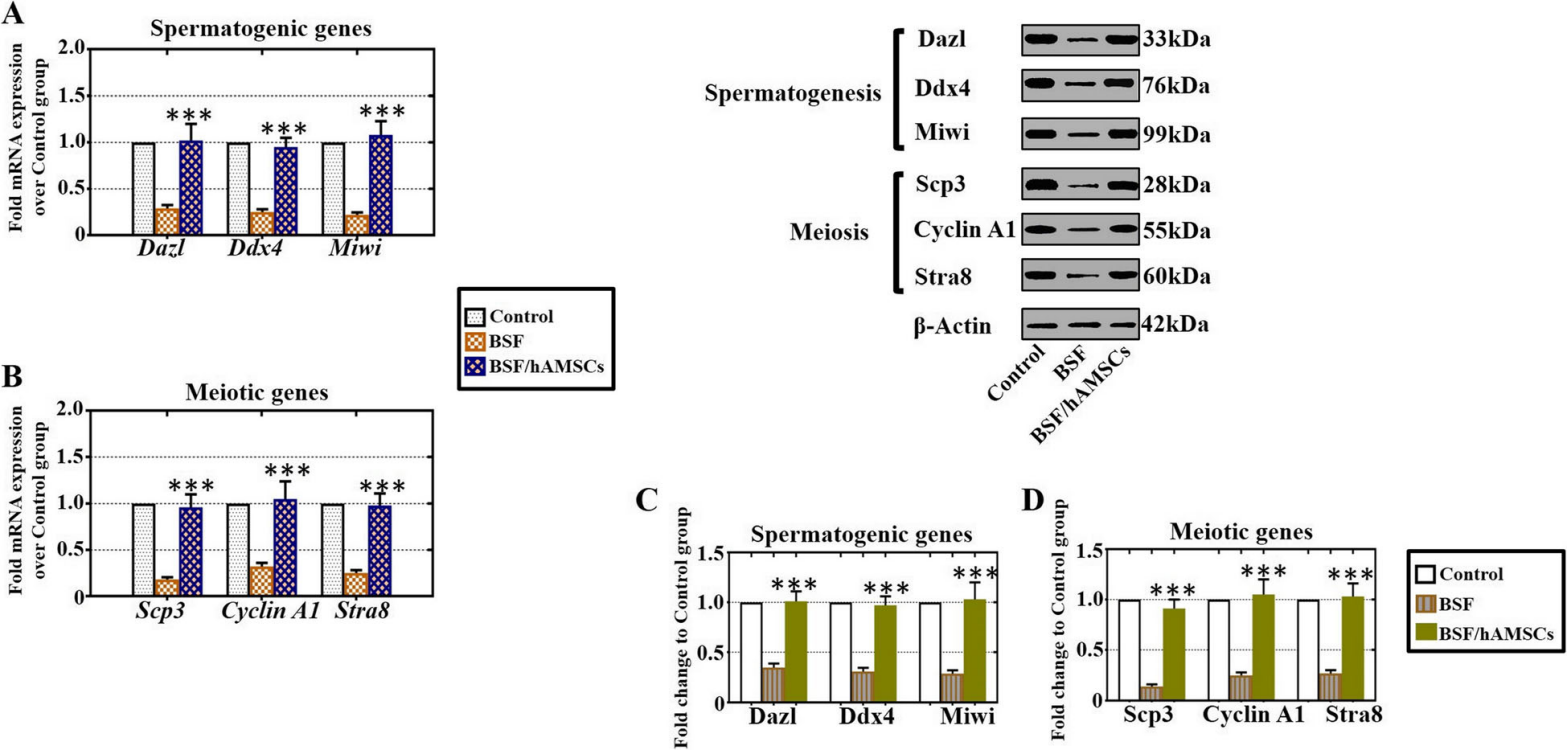
hAMSCs elevated spermatogenesis-associated gene markers in a busulfan-induced testis toxicity mouse model. **a**, **b** The mRNA levels of germ cell-specific genes Dazl, Ddx4, and Miwi and meiosis genes Scp3, Cyclin A1, and Stra8 were examined by qPCR analysis of the three groups. **c**, **d** The protein levels of germ cell-specific genes Dazl, Ddx4, and Miwi and meiosis genes Scp3, Cyclin A1, and Stra8 were inspected by western blot analysis of the three groups. The results are presented as the mean ± SD. ***p < 0.001 (compared to the BSF group). *n* = 10 for each group

To explore the potential degree of spermatogenic function recovery by hAMSCs, the germ cell-specific (GCS) genes Dazl, Ddx4, and Miwi and the meiosis genes Scp3, Cyclin A1, and Stra8 were selected as candidate target genes for analysis. The mRNA and protein expression of these genes was detected by qPCR analysis and western blot. The mRNA levels of the GCS genes Dazl, Ddx4, and Miwi were low in the BSF group, while these GCS genes were highly expressed after hAMSC transplantation (Fig. 7a). Expression of the meiosis genes Scp3, Cyclin A1, and Stra8 was rapidly elevated to normal levels in the BSF/hAMSC group relative to those in the BSF group (Fig. 7b). The protein levels of these GCS genes were obviously suppressed to a lower level in the BSF group, but they recovered to approximately normal levels at 4 weeks after hAMSC transplantation (Fig. 7c). Compared to the BSF group, the BSF/hAMSC group exhibited significant upregulation of the proteins encoded by these meiosis genes (Fig. 7d).

## Discussion

High survival rates are being achieved with the development of cancer therapies, while pre- and postpubertal patients receiving gonadotoxicity therapy may have to confront infertility. Improvements in ART, especially the advent of intracytoplasmic sperm injection (ICSI) in 1992, allow many hopeless, infertile couples due to multiple causes of male infertility to realize parenthood. However, these technologies may transmit potential genetic abnormalities from the father, and these strategies are not suitable for adult men with spermatogenic failure and prepubertal boys [2]. The results of the present research revealed that hAMSC transplantation restored the spermatogenic process in a busulfan-induced testis toxicity mouse model and enhanced proliferation in busulfan-treated mouse Sertoli cells by resisting apoptosis and oxidative stress. To our knowledge, the relationship between hAMSC transplantation and busulfan-induced testis toxicity has not been investigated.

hAMSCs transplanted into a busulfan-induced testis toxicity mouse model restored spermatogenesis, as revealed by histological analysis, with most spermatogonia, spermatocytes, round spermatids, and healthy sperm reappearing in the seminiferous tubules, the number of seminiferous tubules with vacuoles diminishing, and the lumen diameter shrinking (Figs. 1 and 2). The testosterone level was increased after hAMSC transplantation (Fig. 1), which was in accordance with the results of a previous study where BMMSCs were used to treat lead nitrate-induced male infertility in rats [25]. The current investigation also demonstrated that the size and weight of the testes were obviously recovered to normal levels (Fig. 2). In line with our results, ADMSCs transplanted to busulfan-induced azoospermic rats also restored testicular size and weight [12]. Regarding male fertility and clinical practice, semen analysis is used to assess sperm concentration and motility, and it is the cornerstone of diagnosis for various infertility and evaluation for fertility potential in males. Our findings revealed that the total number of sperm and the proportion of rapidly moving sperm were obviously elevated and that the proportion of static sperm was significantly inhibited after hAMSC treatment (Fig. 2). A previous study supported the results that BMMSC treatment for lead nitrate-induced male infertility in rats not only enhanced the total number of sperm and the proportion of motile sperm and morphologically normal sperm but also inhibited the proportion of morphologically abnormal sperm [25]. Recently, another report revealed that human orbital fat tissue-derived MSC transplantation enhanced the sperm number and the percentage of live sperm with progressive motility in rats with testicular torsion [29]. Together, these findings from the testis histology, testicular size and weight, testosterone level, and semen analysis all highlighted that hAMSC treatment could effectively restore endogenous spermatogenesis impaired by busulfan.

Apoptosis is a complex process that tightly regulates the rate of cell division and death and triggers a suicide program with DNA fragmentation enhancing, membrane of nucleus swelling, cytoplasm shrinking, and cell death finally [22]. The findings of our study showed that hAMSCs promoted proliferation and repressed apoptosis in busulfan-damaged Sertoli cells (Fig. 3). Our study also showed that hAMSC transplantation into busulfan-induced impaired testes attenuated apoptosis (Fig. 4). A previous study supported these results and demonstrated that hAMSCs ameliorated cell apoptosis and improved proliferation to restore chronic renal failure [30]. Oxidative stress contributes to DNA damage and results in male infertility [31]. The current experiment showed that the ROS level was obviously decreased with hAMSC treatment in vivo and in vitro (Figs. 5, 6). Consistent with our results, hAMSCs with immunoregulated properties diminished ROS production to decrease neutrophil extracellular traps [32].

Our study showed that the GCS genes Dazl, Ddx4, and Miwi and the meiosis genes Scp3, Cyclin A1, and Stra8 were significantly upregulated not only at the mRNA level but also at the protein level after hAMSC transplantation (Fig. 7). The immunofluorescence staining of SCP3 also increased obviously with hAMSC treatment (Fig. 4). Similar to our results, several studies affirmed that UCMSCs transplanted into mice with azoospermia induced by busulfan resumed the expression of Miwi, Vasa, and Scp3 [10], and exosomes of urine-derived stem cells transplanted into nonobstructive azoospermia mice facilitated spermatogenesis by enhancing the expression of Pou5f1, Prm1, Sycp3, and Dazl [14].

## Conclusion

In conclusion, the therapeutic effect of hAMSCs on endogenous spermatogenesis was confirmed in this study. Furthermore, the underlying mechanisms were also explained. hAMSCs are required for resisting apoptosis by upregulating the proliferation marker Ki67 and the anti-apoptotic markers BCL2 and SURVIVIN and by downregulating markers of apoptosis, TUNEL, Annexin V, CASPASE3, and CASPASE9. In addition, hAMSCs are associated with repressing oxidative stress by decreasing ROS, LDH, and MDA expression and increasing GR, SOD, GPx, and CAT expression. Taken together, these discoveries reveal a vital role for hAMSCs in resisting apoptosis and oxidative stress to enable male fertility preservation that was impaired by busulfan.

## Supplementary information

**Additional file 1: Table S1.** Designations, sequences, and the sizes of real-time PCR amplicons.

## Data Availability

All the data generated or analyzed during this study are included in this published article.

## Abbreviations

ART: Assisted reproductive technology
ICSI: Intracytoplasmic sperm injection
MSC: Mesenchymal stem cell
hAMSCs: Human amnion mesenchymal stem cells
UCMSCs: Umbilical cord mesenchymal stem cells
BMMSCs: Bone marrow mesenchymal stem cells
ADMSCs: Adipose tissue mesenchymal stem cells
BSF: Busulfan
GCS: Germ cell-specific
OS: Oxidative stress
ROS: Reactive oxygen species
MDA: Malondialdehyde
SOD: Superoxide dismutase
CAT: Catalase
LDH: Lactate dehydrogenase
GR: Glutathione reductase
GPx: Glutathione peroxidase
ELISA: Enzyme-linked immunosorbent assay
FACS: Fluorescence-activated cell sorting
HE: Hematoxylin and eosin
qPCR: Quantitative polymerase chain reaction
CASA: Computer-assisted semen analysis
GAPDH: Glyceraldehyde 3-phosphate dehydrogenase
