# Supplementary material for: Human amnion mesenchymal stem cells restore spermatogenesis in mice with busulfan-induced testis toxicity by inhibiting apoptosis and oxidative stress
Source: Stem Cell Res Ther. 2020 Jul 16;11:290. doi: 10.1186/s13287-020-01803-7 (PMC7367397; doi:10.1186/s13287-020-01803-7)
Supplement: Supplementary file 1 — Additional file 1: Table S1. Designations, sequences, and the sizes of real-time PCR amplicons. [file 13287_2020_1803_MOESM1_ESM.doc]

**Table S1 Designations, sequences, and the sizes of real-time PCR amplicons**

| **Name** | **Sequence from 5'-3'** | **Size (bp)** |
| --- | --- | --- |
| Dazl (M) Fw | GCTGACGTGCTTTGATTTTTGT | 117 |
| Dazl (M) Rev | GCTGACGTGCTTTGATTTTTGT |
| Ddx4 (M) Fw | CGGAGAGGAACCTGAAGCTA | 164 |
| Ddx4 (M) Rev | ACTCGCCAATATCTGATGAAGC |
| Miwi (M) Fw | CCGTTAACCCAGAGGAGTGAC | 199 |
| Miwi (M) Rev | GGTGGGATGTACCCAGGTTG |
| Scp3 (M) Fw | ACTTTAGTGGCGGGAAGACG | 230 |
| Scp3 (M) Rev | CCAGGTGCTTTTGCTTGACC |
| Cyclin A1 (M) Fw | TTCTGGTTTGACTCCCGGAC | 183 |
| Cyclin A1 (M) Rev | AAATCGGCTGACCACTCTGG |
| Stra8 (M) Fw | AGGGGTGTAAGAACTGGCG | 254 |
| Stra8 (M) Rev | GACTGCCCGTCGCAGAATAA |
| GAPDH (M) Fw | TTCCAGTATGACTCTACCCACGGCA | 137 |
| GAPDH (M) Rev | GCACCAGCATCACCCCATTTG |

**M=Mouse.**
